# Characterization of Ca_V_1.2 exon 33 heterozygous knockout mice and negative correlation between *Rbfox1* and Ca_V_1.2 exon 33 expressions in human heart failure

**DOI:** 10.1080/19336950.2017.1381805

**Published:** 2017-09-26

**Authors:** Juejin Wang, Guang Li, Dejie Yu, Yuk Peng Wong, Tan Fong Yong, Mui Cheng Liang, Ping Liao, Roger Foo, Uta C. Hoppe, Tuck Wah Soong

**Affiliations:** a Department of Physiology, National University of Singapore, Singapore; b Department of Physiology, Nanjing Medical University, Nanjing, P.R. China; c Key Laboratory of Medical Electrophysiology, Ministry of Education, Institute of Cardiovascular Research, Southwest Medical University, Luzhou, P.R. China; d National Neuroscience Institute, Singapore; e Genome Institute of Singapore, Singapore; f Department of Internal Medicine II, Paracelsus Medical University, Salzburg, Austria; g Graduate School for Integrative Sciences and Engineering, National University of Singapore, Singapore; h Neurobiology/Ageing Programme, National University of Singapore, Singapore

**Keywords:** alternative splicing, cardiomyopathy, Ca_V_1.2 calcium channel, heterozygous knockout, Rbfox

## Abstract

Recently, we reported that homozygous deletion of alternative exon 33 of Ca_V_1.2 calcium channel in the mouse resulted in ventricular arrhythmias arising from increased Ca_V_1.2_Δ33_ *I_CaL_* current density in the cardiomyocytes. We wondered whether heterozygous deletion of exon 33 might produce cardiac phenotype in a dose-dependent manner, and whether the expression levels of RNA splicing factors known to regulate alternative splicing of exon 33 might change in human heart failure. Unexpectedly, we found that exon 33^+/−^ cardiomyocytes showed similar Ca_V_1.2 channel properties as wild-type cardiomyocyte, even though Ca_V_1.2_Δ33_ channels exhibit a gain-in-function. In human hearts, we found that the mRNA level of splicing factor *Rbfox1*, but not *Rbfox2*, was downregulated in dilated cardiomyopathy, and *CACNA1C* mRNA level was dramatically decreased in the both of dilated and ischemic cardiomyopathy. These data imply *Rbfox1* may be involved in the development of cardiomyopathies via regulating the alternative splicing of Ca_V_1.2 exon 33. (149 words)

## Introduction

Ca_V_1.2 L-type calcium channels have essential roles in the cardiac excitation-contraction coupling and development. Alternative splicing in *CACNA1C*, encoding the Ca_V_1.2 pore-forming subunit α1_C_, modulates the function of Ca_V_1.2 calcium channels. Exon 33 has been identified in human and rodent Ca_V_1.2 α1_C_ subunit by transcript-scanning;^1,2^ this alternative exon has been reported to affect the biochemical and biophysical functions of heterologously expressed Ca_V_1.2 channels.^1–6^ Recently, we generated Ca_V_1.2 exon 33 specific knockout mice (exon 33^−/−^) and these exon 33-null mice were found to develop cardiac arrhythmia and dysfunction, owing to increased Ca_V_1.2 channel currents arising from a leftward shift of voltage-dependent activation and inactivation potentials as compared to wild-type (WT) (exon 33^+/+^) cardiomyocytes.^7^ Thus, we asked whether cardiomyocytes isolated from heterozygous exon 33-knockout mice might produce intermediate changes in biophysical properties of the Ca_V_1.2 channels and how this might affect excitability of the cardiomyocytes.

In our previous work, we found that the expression of Ca_V_1.2 alternative exon 33 was significantly increased in human failing hearts,^7^ and these data raise a potential relevance to clinical management of heart failure. A second question we asked was the mechanism by which alternative splicing of exon 33 of Ca_V_1.2 channels might be regulated in the heart. The (U)GCAUG elements, which can be recognized and bound by RNA binding protein *Rbfox1/2*, were identified in the intronic sequence surrounding Ca_V_1.2 exon 33.^8^ Functionally, *Rbfox1/2* could enhance the inclusion of alternative exon 33 of Ca_V_1.2 calcium channels.^8,9^ To date, *Rbfox1/2* was reported to be crucial in cardiac development and different cardiomyopathies via regulation of serial splicing events,^10–14^ indicating a plausible role in the regulation of alternative exon 33 of Ca_V_1.2 calcium channels in the heart. In this follow-up study of our previous work, we measured *Rbfox1/2* mRNA levels in human failing and non-failing heart samples, in order to explore the possible association between the expressions of *Rbfox* and exon 33 of Ca_V_1.2 in cardiomyopathies.

## Results

### Exon 33^+/−^ cardiomyocyte has similar Ca_V_1.2 channel properties with WT cardiomyocyte

**Figure 1.**
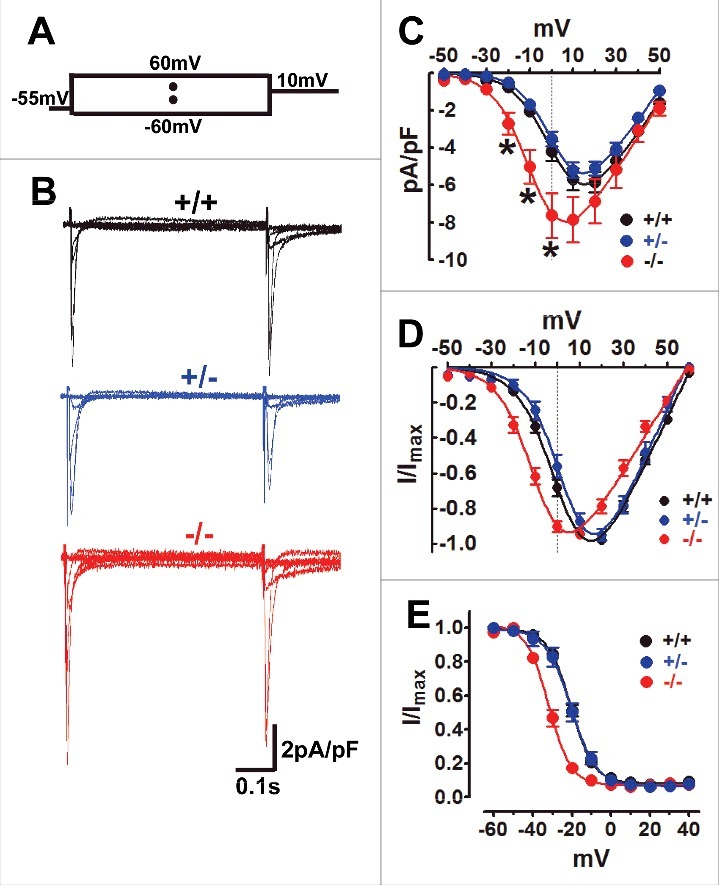
Exon 33^+/−^ cardiomyocyte has similar Ca_V_1.2 channel properties with WT cardiomyocyte. (A) The stimulus waveform was used to induce *I*_Ca_ of cardiomyocyte, briefly *I*_Ca_ was recorded under the different testing potentials, increased from −60 mV to 60 mV (10 mV increase each step) followed by 10 mV depolarizing-pulse to induce steady-state inactivation in cardiomyocytes when using the 1.8 mmol/L Ca^2+^ as charger carrier. (B) Exemplary current traces were recorded from WT (black), exon 33^+/−^ (blue) or exon 33^−/−^ (red) cardiomyocytes. (C) Current densities of Ca_V_1.2 channels in WT (n = 11 cells, *V*_0.5_ = 2.82±2.3 mV), exon 33^+/−^ (n = 20 cells, *V*_0.5_ = 3.18±1.7 mV) and exon 33^−/−^ (n = 18 cells, *V*_0.5_ = −7.28±3.3 mV) cardiomyocytes (*P<0.05 vs. WT, unpaired *t* test). (D) Normalized current-voltage relationships of Ca_V_1.2 channels in WT, exon 33^+/−^ and exon 33^−/−^ cardiomyocytes. (E) Steady-state inactivation of Ca_V_1.2 channels in WT (n = 16 cells, *V*_0.5,inact_ = −20.63±0.44 mV), exon 33^+/−^ (n = 14 cells, *V*_0.5,inact_ = −20.46±0.85 mV) and exon 33^−/−^ (n = 12 cells, *V*_0.5,inact_ = −31.94±0.64 mV) cardiomyocytes.

In the exon 33^+/−^ ventricular tissue, the upper band density of Ca_V_1.2 channels with inclusion of exon 33 (Ca_V_1.2_33_) is similar with the lower band, which are the Ca_V_1.2 channels with exon 33 skipping (Ca_V_1.2_Δ33_),^7^ indicating ∼50% Ca_V_1.2_Δ33_ channels in exon 33^+/−^ hearts, compared with ∼7.6% Ca_V_1.2_Δ33_ channels in WT hearts, and 100% Ca_V_1.2_Δ33_ channels in exon 33^−/−^ hearts.^7^ To investigate the Ca_V_1.2 current properties of the exon 33^+/−^ cardiomyocytes, we performed voltage-clamp recordings in isolated ventricular myocytes. Unexpectedly, the current density of exon 33^+/−^ cardiomyocyte is almost same with WT cardiomyocyte, but much smaller than exon 33^−/−^ cardiomyocyte (Fig. 1A–C). Moreover, the current-voltage (*I*-*V*) relationship curve (Fig. 1D) and steady-state inactivation potential (Fig. 1E) of exon 33^+/−^ cardiomyocyte were also similar with WT cardiomyocyte, but rightward shifted compared with exon 33^−/−^ cardiomyocyte. Taken together, our data indicated that heterozygous deletion of alternative exon 33 produced half the Δexon33-containing Ca_V_1.2 transcripts but functionally the biophysical properties of the Ca_V_1.2 Ca^2+^ currents recorded in Ca_V_1.2 exon 33^+/−^ cardiomyocytes were similar to WT. As we could not raise an antibody to specifically detect exon 33, we are unable to determine the levels of surface expressions of Ca_V_1.2_33_ or Ca_V_1.2_Δ33_ channels.

### Exon 33^+/−^ cardiomyocyte shows normal membrane excitation

**Figure 2.**
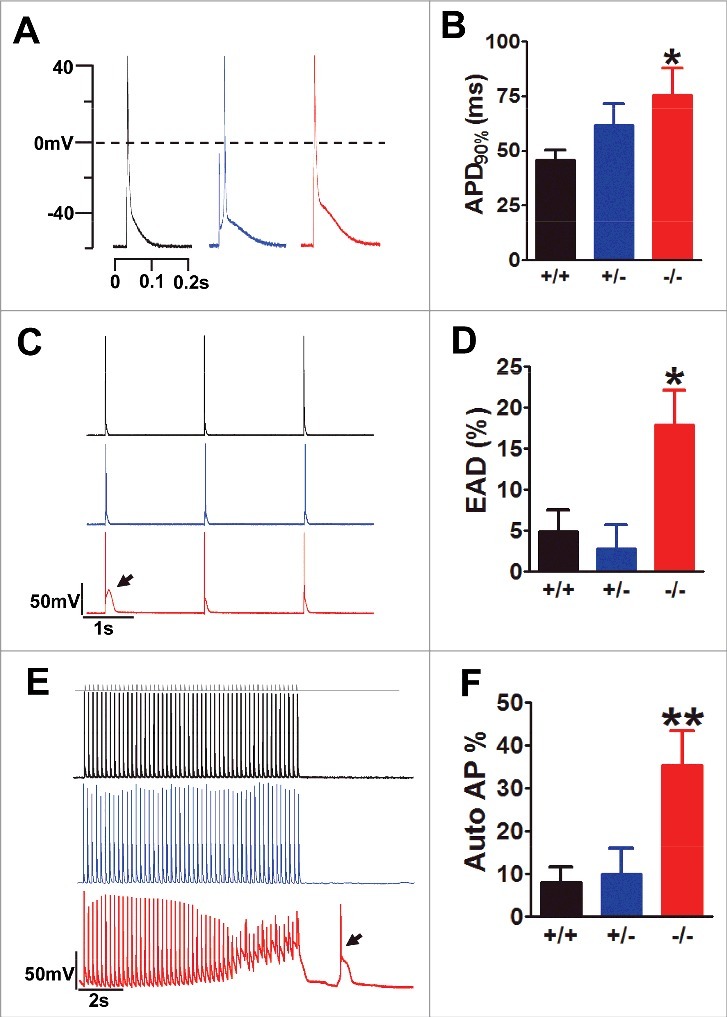
Exon 33^+/−^ cardiomyocyte did not show any signs of abnormal excitations. (A) AP waveforms recorded from WT (black), exon 33^+/−^ (blue) and exon 33^−/−^ (red) cardiomyocytes. (B) APD after 90% of repolarization (APD_90%_) from WT (n = 11 cells), exon 33^+/−^ (n = 14 cells) and exon 33^−/−^ (n = 16 cells) (*P<0.05 vs. WT, unpaired *t* test). (C and D) Detection of EADs in cardiomyocytes of WT (n = 73 cells, 17 mice), exon 33^+/−^ (n = 24 cells, 5 mice) or exon 33^−/−^ mice (n = 73 cells, 16 mice) at a 0.5-Hz pacing rate. (*P* = 0.0139 one-way ANOVA; *P<0.05 vs. WT, Bonferroni *post hoc* test). (E and F) Detection of autonomous APs in cardiomyocytes from WT (n = 73 cells, 17 mice), exon 33^+/−^ (n = 24 cells, 5 mice) and exon 33^−/−^ (n = 73 cells, 16 mice) a 5-Hz pacing rate (*P* = 0.0052, one-way ANOVA; **P<0.01 vs. WT, Bonferroni *post hoc* test).

Further, we explored the properties of cardiomyocyte excitation under current-clamp recording. Unlike exon 33^−/−^ cardiomyocyte, the action potential duration (APD) after 90% repolarization (APD_90%_) of exon 33^+/−^ cardiomyocyte was not increased but was similar to WT cardiomyocyte (Fig. 2A–B). This result could be explained by the similar characterized current properties between exon 33^+/−^ and WT cardiomyocyte (Fig. 1C–E). Following that, we also measured the early after-depolarization (EAD) and autonomous action potentials (APs), two hallmarks of cardiac arrhythmias.^15,16^ Our data showed the occurrence of EAD in exon 33^+/−^ cardiomyocytes (∼3%) had no differences as compared with WT cardiomyocytes (∼5%), while exon 33^−/−^ cardiomyocytes had a significant increase of EAD occurrence (∼18%) (Fig. 2C–D). Another indicator of cardiac arrhythmia is the generation of autonomous APs after the cessation of electrical stimulation, exon 33^−/−^ cardiomyocytes produced much higher frequency of autonomous APs (∼36%) as previously indicated,^7^ but exon 33^+/−^ cardiomyocytes showed the similar low frequency of autonomous APs as compared with WT cardiomyocytes (Fig. 2E–F). In sum, exon 33^+/−^ cardiomyocytes did not show any signs of abnormal membrane excitations, such as EAD and autonomous APs, indicating heterozygous knockout of alternative exon 33 of Ca_V_1.2 calcium channels might be not contribute to cardiac arrhythmia.

### Rbfox1 expression is correlated with exon 33 and CACNA1C mRNA levels in human hearts

**Figure 3.**
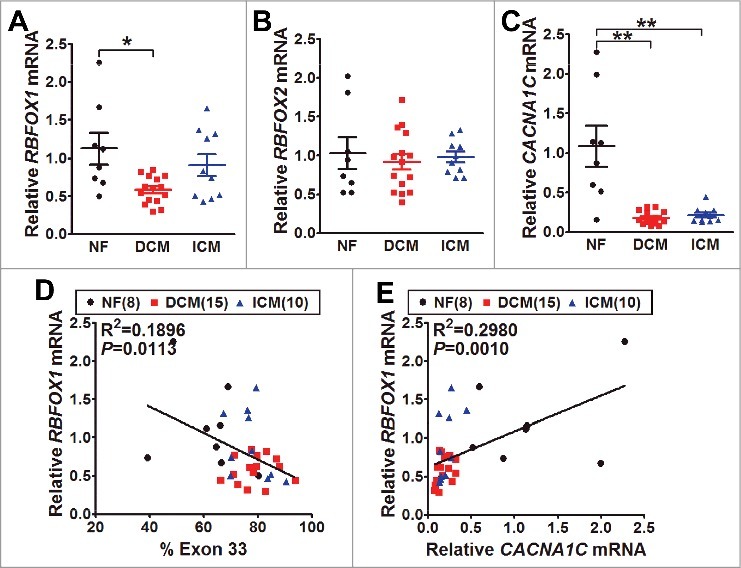
The mRNA expression of *Rbfox1/2* and Ca_V_1.2 α1_C_ in human hearts. (A) The mRNA levels of *Rbfox1* were measured by real-time RT-PCR from the samples of human normal (NF, black, n = 8), dilated cardiomyopathy (DCM, red, n = 15) and ischemic cardiomyopathy (ICM, blue, n = 10) hearts. The relative expression was normalized by internal expression of human *GAPDH* and *RPLPO* mRNA (*P* = 0.0103, one-way ANOVA; *P<0.05 vs. WT, Bonferroni *post hoc* test). (B) The mRNA levels of *Rbfox2* were also measured by real-time RT-PCR from NF (n = 8), DCM (n = 15) and ICM (n = 10) hearts. (C) Ca_V_1.2 α1_C_ mRNA expression was measured by real-time RT-PCR from NF (n = 8), DCM (n = 15) and ICM (n = 10) hearts (**P<0.01, one-way ANOVA; **P<0.01 vs. WT, Bonferroni *post hoc* test). The correlations between alternative exon 33 expression and *Rbfox1* (D) or Ca_V_1.2 α1_C_ (E) mRNA level in human hearts were analyzed by linear regression, R^2^ refers to the coefficient of determination.

To address the possible pathological relevance, we collected human non-failing and failing heart samples^7^ to measure *RBFOX1/2* and *CACNA1C* mRNA expression. Specifically, *Rbfox1* expression in dilated cardiomyopathy (DCM) failing hearts was significantly lower than non-failing hearts (Fig. 3A). However, *Rbfox2* expression had no significant differences among non-failing, dilated and ischemic cardiomyopathy (ICM) failing hearts (Fig. 3B). Moreover, *CACNA1C* mRNA expression was dramatically downregulated in both dilated and ischemic cardiomyopathy failing hearts (Fig. 3C), implying that decreased Ca_V_1.2 calcium channel may also be involved in the induction of hypertrophy and/or heart failure.^17^ More interestingly, we found that *Rbfox1* expression was negatively correlated with exon 33 inclusion (Fig. 3D), but positively correlated with *CACNA1C* mRNA expression in human hearts (Fig. 3E), these suggest dysregulated *Rbfox1* might play some roles in the regulation of human Ca_V_1.2 expression. Therefore, it is reasonable to believe that altered *Rbfox1* expression may contribute to the changes in expression and alternative splicing of Ca_V_1.2 calcium channels that led to lower channel activity in failing hearts.

## Discussion

Alternative splicing of Ca_V_1.2 calcium channel is recognized as an important post-transcriptional modification that provides modulation of channel function. Our previous work has directly addressed the *in vivo* significance of altered Ca_V_1.2 calcium channel property arising from alternative splicing of exon 33 that showed the deletion of exon 33 induced enhanced channel activity and abnormal cardiomyocyte excitation. This process in turn resulted in ventricular arrhythmia and cardiac dysfunction in mice.^7^ In normal rodent hearts, the proportion of Ca_V_1.2 calcium channels with inclusion of exon 33 is more than 90%.^2-4,7^ As such, what could the phenotype of the heart be if this proportion decreases to ∼50%? Here, we investigated that 50% expression at the transcript level of exon 33 in Ca_V_1.2 channel in heterozygous knockout mice did not produce any significant changes in channel properties and therefore not surprisingly no gross differences in cardiac electrical properties in cardiomyocytes. Our data raised the question on how the presence of Ca_V_1.2_33_ could completely override the contribution of Ca_V_1.2_Δ33_ channels in cardiomyocytes. There are several possibilities to explain this phenotype. First, Ca_V_1.2 channels with exon 33 (Ca_V_1.2_33_) make a chief role in the excitation-contraction coupling in the normal heart, and a small portion of Ca_V_1.2_33_ channels is sufficient to maintain the regular activities of Ca_V_1.2 channels. Second, Ca_V_1.2 α1_C_ subunits are known to be bound with Ca_V_β subunits to traffic them to cell membrane;^18^ though Ca_V_1.2_Δ33_ channels are transcribed in cardiomyocytes, the trafficking of these Ca_V_1.2_Δ33_ channels might be disturbed when competing with Ca_V_1.2_33_ channels, thus reducing the contribution of Ca_V_1.2_Δ33_ channels on the cell surface in heterozygous knockout cardiomyocytes. Third, as oligomerization of Ca_V_1.2 channels can form a “coupled gating” function in excitable cells,^19,20^ Ca_V_1.2 channels might have a “selective ability” to form the oligomerization with neighboring Ca_V_1.2_33_ channels, but not Ca_V_1.2_Δ33_ channels in cardiomyocyte; thus the exclusion of alternative exon 33 may affect the oligomerization of Ca_V_1.2 channels. Nevertheless, the detailed mechanisms of Ca_V_1.2_33_-Ca_V_1.2_Δ33_ channel interaction are warranted for further investigation.

Another issue is which mechanism(s) regulates the specific expression pattern of alternative exon 33 of Ca_V_1.2 calcium channels in human hearts. The possible candidate is *Rbfox1/2*, which belong to the RNA-binding proteins,^21^ it has been known to directly enhance the inclusion of alternative exon 33 of Ca_V_1.2 channel during neuronal development.^8^ Therefore, what is the relevance of *Rbfox1/2* in the pathology of cardiac diseases? In this work, we found the expression of *Rbfox1* was dramatically decreased in human DCM when compared with non-failing hearts, which is consistent with Gao's report in which they indicated the expression of *Rbfox1* was markedly diminished in human DCM and in transverse aortic restriction-induced murine heart failure.^12^ These imply *Rbfox1* indeed takes part in the alternative splicing regulation of diseased hearts. Although *Rbfox1* was reported to enhance the inclusion of Ca_V_1.2 alternative exon 33 in rodent^8^, in contrast we found the expression of *Rbfox1* was negatively correlated with the expression of exon 33 in human hearts. This result could be considered as the increased expression of Ca_V_1.2_33_ channels could be a compensatory response to heart failure in humans, but we cannot exclude other potential splicing factors that might directly or indirectly regulate the alternative splicing of Ca_V_1.2 exon 33 in human heart. Here, we also found the dramatic decrease of Ca_V_1.2 α1_C_ mRNA in human DCM and ICM hearts, in line with the reduced activities of Ca_V_1.2 channels in human failing hearts^22^. Moreover, the expression of *Rbfox1* was positively correlated with the expression of Ca_V_1.2 calcium channels in human hearts by our study. Therefore, induction of *Rbfox1* expression might have some beneficial effects on the cardiomyopathies.^12^

In conclusion, 50% loss of alternative exon 33 in Ca_V_1.2 calcium channel did not affect the electrophysiological properties of Ca_V_1.2 channels recorded in mouse cardiomyocytes, and *Rbfox1* might be a modulator of exon 33 alternative splicing in human heart. Nevertheless, further studies are required to clarify the molecular mechanisms how the presence of exon 33 affect the structure and function of Ca_V_1.2 channels and what upstream signals may be important to regulate *Rbfox* expression.

## Materials and methods

### Human samples and animals

Human heart samples and knockout mice of alternative exon 33 of Ca_V_1.2 (exon 33^−/−^) were obtained or generated as described previously.^7^ Exon 33^−/−^ mice were crossed with C57BL/6J mice to generate heterozygous knockout mice (exon 33^+/−^). The genotype was determined by PCR method. Isolation of cardiomyocytes was also described in our previous report.^7^ All animals were treated ethically in accordance with approved institutional IACUC protocol of the National University of Singapore.

### RT-PCR

Total RNA was extracted using Trizol (Invitrogen) as indicated in manufacturer's protocol. Reverse transcription was performed using Superscript III (Invitrogen). Real-time PCR was used in quantitative analysis of the *Rbfox1/2* and Ca_V_1.2 channel mRNA expression level. FAM labeled *CACNA1C* probe located at the junction of constitutive exon 3 and exon 4 (Assay ID Hs00167681_m1, Applied Biosystems), and *RBFOX1/2* (Assay ID Hs01125659_m1 and Hs00204814_m1, Applied Biosystems) were used to measure the standard mRNA expressions. Human *GAPDH* (Assay ID 4333764T, Applied Biosystems) and *RPLPO* (large ribosomal protein) (Assay ID 4333761T, Applied Biosystems) were used as endogenous control (FAM™ Dye/MGB Probe, Applied Biosystems).

### Electrophysiology

Whole-cell L-type calcium current and action potentials of cardiomyocytes were recorded using the patch-clamp technique as previously described.^7^

### Statistical analysis

Data is reported as mean ± S.E.M. Statistical significance was analyzed using a student *t* test, or one-way ANOVA followed by *post hoc* multiple comparisons test. The relationships between *Rbfox1* expression and exon 33 or Ca_V_1.2 expression were analyzed by linear regression. A value of *P* < 0.05 was considered as statistical significance.
